# Percolation Magnetism in Ferroelectric Nanoparticles

**DOI:** 10.1186/s11671-017-2146-1

**Published:** 2017-06-02

**Authors:** Iryna S. Golovina, Serhii V. Lemishko, Anna N. Morozovska

**Affiliations:** 1 Institute of Semiconductor Physics, National Academy of Sciences of Ukraine, pr. Nauky 41, Kyiv, 03028 Ukraine; 2 Department of Materials Science and Engineering, Drexel University, Philadelphia, PA 19104 USA; 3 Institute of Physics, National Academy of Sciences of Ukraine, pr. Nauky 46, Kyiv, 03028 Ukraine

**Keywords:** KTaO_3_, KNbO_3_, Ferroelectric nanoparticles, Percolation magnetism, Modeling

## Abstract

Nanoparticles of potassium tantalate (KTaO_3_) and potassium niobate (KNbO_3_) were synthesized by oxidation of metallic tantalum in molten potassium nitrate with the addition of potassium hydroxide. Magnetization curves obtained on these ferroelectric nanoparticles exhibit a weak ferromagnetism, while these compounds are nonmagnetic in a bulk. The experimental data are used as a start point for theoretical calculations. We consider a microscopic mechanism that leads to the emerging of a ferromagnetic ordering in ferroelectric nanoparticles. Our approach is based on the percolation of magnetic polarons assuming the dominant role of the oxygen vacancies. It describes the formation of surface magnetic polarons, in which an exchange interaction between electrons trapped in oxygen vacancies is mediated by magnetic impurity Fe^3+^ ions. The dependences of percolation radius on concentration of the oxygen vacancies and magnetic defects are determined in the framework of percolation theory.

## Background

Fabrication and intense studies of nanoparticles from dielectric oxides, which are nonmagnetic in a bulk, revealed the ferromagnetism in them, e.g., in HfO_2_ [1], TiO_2_ [2–4], ZnO [5–7], SnO_2_ [7, 8], KTaO_3_ [9], and KNbO_3_ [10]. Different origins of the phenomena were considered, such as metallic clusters, secondary phases, bound magnetic polarons, charge carriers, and oxygen vacancies [2, 3, 5, 8–13]. The latter ones are hybridized in vicinity of nanoparticle surface, thereby acquiring magnetic properties [12, 13]. Currently, the explanations are still controversial and there is no consensus on the source of ferromagnetism. Therefore, research activity is continuing in the direction.

Among ferroelectrics, recently, in nanocrystals (average particle size is 80 nm) of potassium tantalate and potassium niobate, produced by the novel technology of the metal (Ta or Nb) oxygenation in molten potassium nitrate [14], magnetic resonance and static magnetization methods established experimentally the appearance of ferromagnetic subsystem along with a paramagnetic subsystem [10, 15, 16]. This effect is absent in larger crystals (with sizes >200 nm) of the compounds obtained by the same technology. The compounds are nonmagnetic in the bulk. The intentional doping of potassium tantalate (KNbO_3_) and potassium niobate (KTaO_3_) nanocrystals by iron and manganese separately resulted in the increase of the paramagnetic component, while the ferromagnetic subsystem remains unchanged [15, 16]. It has been suggested that the reason for the appearance of the magnetic properties of pristine ferroelectric nanocrystals are magnetic defects, which can be both iron impurity atoms, forming metallic clusters on the surface of the nanoparticles, and oxygen vacancies [9]. In addition to the abovementioned experimental methods, this assumption was based on the data of elemental analysis and theoretical estimates.

In order to determine the microscopic mechanism of the observed phenomenon, in this work, we analyze the situation using a percolation theory. Percolation theory described quite well a number of effects in disordered magnetic systems, earlier in ferrodielectrics [17], later on in dilute magnetic semiconductors (e.g., [18–21]). Magnetic subsystem is treated as a bound magnetic polaron in dilute semiconductors. This model was firstly proposed by the authors of [22] and subsequently developed by the authors of Refs. [11, 23].

Assuming the dominant role of oxygen vacancies, we use the model of bound magnetic polarons and find the percolation radius at which the exchange interactions between electrons trapped in oxygen vacancies mediated by magnetic impurity ions induce a surface ferromagnetic ordering in ferroelectric KTaO_3_ and KNbO_3_ nanoparticles.

### Experimental Data and Model of Ferromagnetic Ordering

In order to justify the proposed model of ferromagnetic ordering, at first, we expound the experimental data obtained on the ferroelectric nanoparticles КTaO_3_ (KTO) and KNbO_3_ (KNO).

**Fig. 1:**
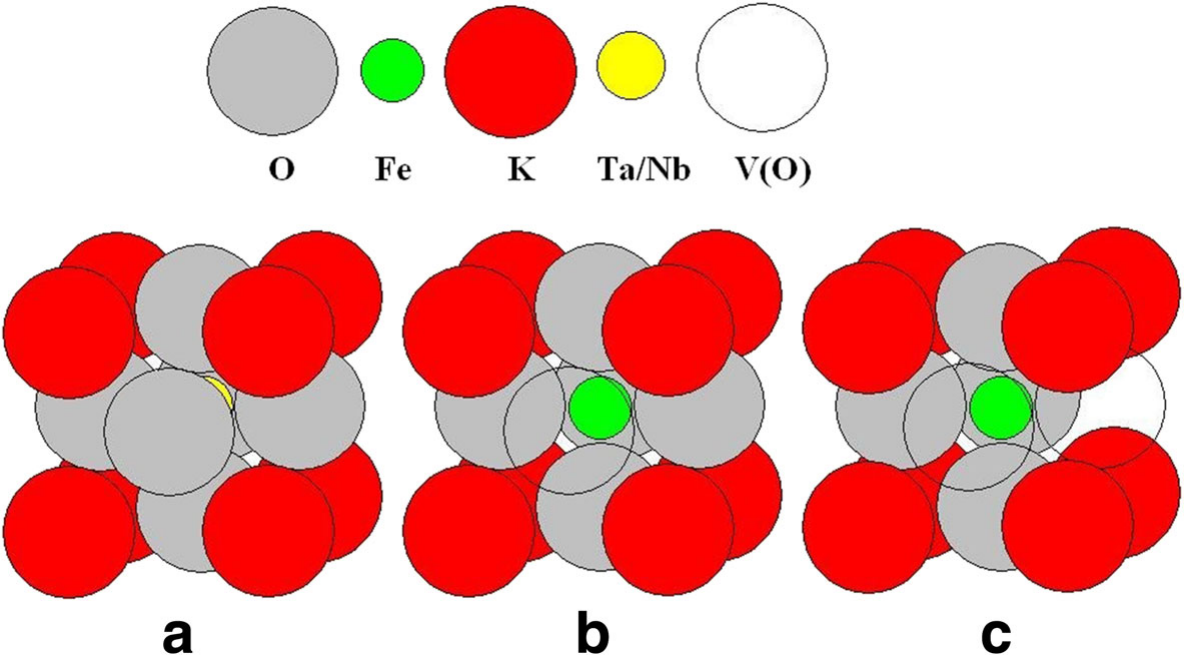
Models of low-symmetric paramagnetic centers of Fe^3+^ in KTaO_3_ and KNbO_3_ nanocrystals. **a** Unit cell without defects, **b** axial Fe^3+^-V(O) center, and **c** rhombic Fe^3+^-2 V(O) center

Examined ferroelectric nanoparticles are nominally pure, i.e., no dopants were specially incorporated. However, elemental analysis performed with a Shimadzu ICPE-9000 inductively coupled plasma atomic emission spectrometer (ICP-AES) shows that Fe is present in both materials as unavoidable impurity in the amount of 0.06 mol.% in КTaO_3_ and 0.008 mol.% in KNbO_3_. It is also known that the oxygen vacancies always exist in oxide ferroelectrics, such as the perovskite-type (general formula ABO_3_), resulting in some degree of non-stoichiometry in these compounds. It has been shown experimentally that two magnetic subsystems (paramagnetic and ferromagnetic) are present in nanocrystals KTaO_3_ and KNbO_3_ [9, 10, 15]. Paramagnetic subsystem consists of separate noninteracting magnetic Fe^3+^ ions in KTaO_3_, and Fe^3+^ and Mn^2+^ ions in KNbO_3_. Moreover, the structure of the paramagnetic center, as determined from electron paramagnetic resonance (EPR) measurements, includes an oxygen vacancy V(O), which lowers the center symmetry [9, 15]. For illustrative purposes, two types of paramagnetic centers, of axial and rhombic symmetry, in which Fe^3+^ ion replaces Ta^5+^ (or Nb^5+^) ion, are shown in Fig. 1. To reach the charge compensation, one (axial center) or two (rhombic center) oxygen vacancies V(O) are formed in the structure of these centers.

As suggested in the papers [9, 15], ferromagnetic subsystem is a surface cluster(s) consisting of iron atoms and oxygen vacancies. Thermal annealing in an oxygen atmosphere, carried out in the paper [9], shows that oxygen vacancies definitely contribute to the formation of ferromagnetism in these compounds. On one side, the vacancies hybridize and become magnetic close to nanoparticles’ surface [13]. On the other side, electrons which inherently emerge in non-stoichiometric oxides are often trapped by oxygen vacancies. In both cases, the oxygen vacancy has spin *S* = ½ and bears corresponding magnetic moment.

**Fig. 2:**
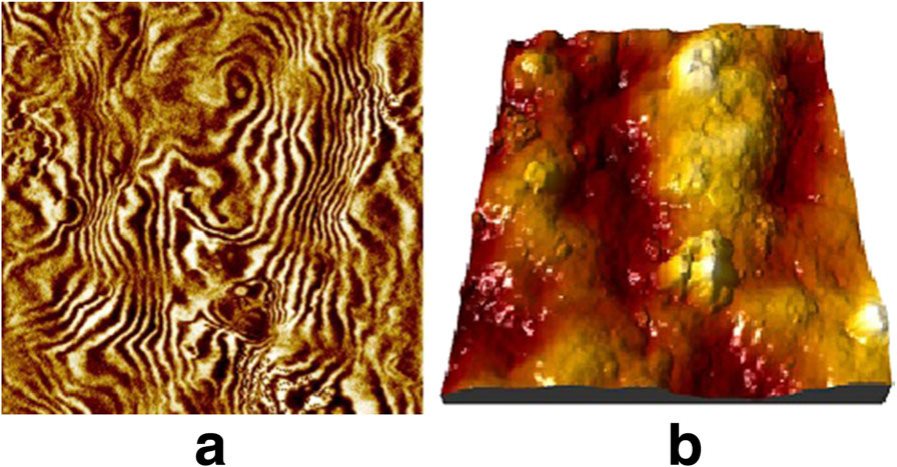
Surface images with magnetized regions in the KTaO_3_ nanocrystals. **a** Magnetic field gradient mapping and **b** surface relief image

The presence of surface ferromagnetism has been confirmed experimentally by atomic force microscopy (AFM) in a magnetic field gradient mode. For example, Fig. 2 shows the surface images obtained for the sample of nanocrystalline KTaO_3_. Magnetic force microscopy (MFM) measurements were performed by Dimension 3000 NanoScope IIIa scanning probe microscope for mapping the spatial variation of the magnetization structure of the out-of-plane component of the magnetic stray field of the KTaO_3_ sample surface. Magnetic force gradients were measured using two-pass technique (lift mode) where topography was scanned at the first pass in the tapping mode and then the magnetic field gradients were mapped at the second one using oscillation frequency shift of the probe moving over surface (lift height was 300 nm). The cobalt-coated Veeco magnetic force probes (MESP) with coercivity of ~400 Oe, magnetic moment of the 1 · 10^−13^ emu and 25-nm nominal tip apex radius were used. Before measurements, the probe was magnetized using a strong permanent magnet with the field aligned along the tip vertical axis.

Also, the static magnetization loops have been obtained experimentally at two temperatures for each compound, at 290 and 150 K for KTaO_3_ and at 290 and 110 K for KNbO_3_. The experimental magnetization curves are described by the formula:

1 $$ M\approx {M}_S{ \tanh}^{-1}\left(\frac{H{ V}_0{M}_S}{k_B T}\right)-\frac{k_B T}{H{ V}_0} $$

**Fig. 3:**
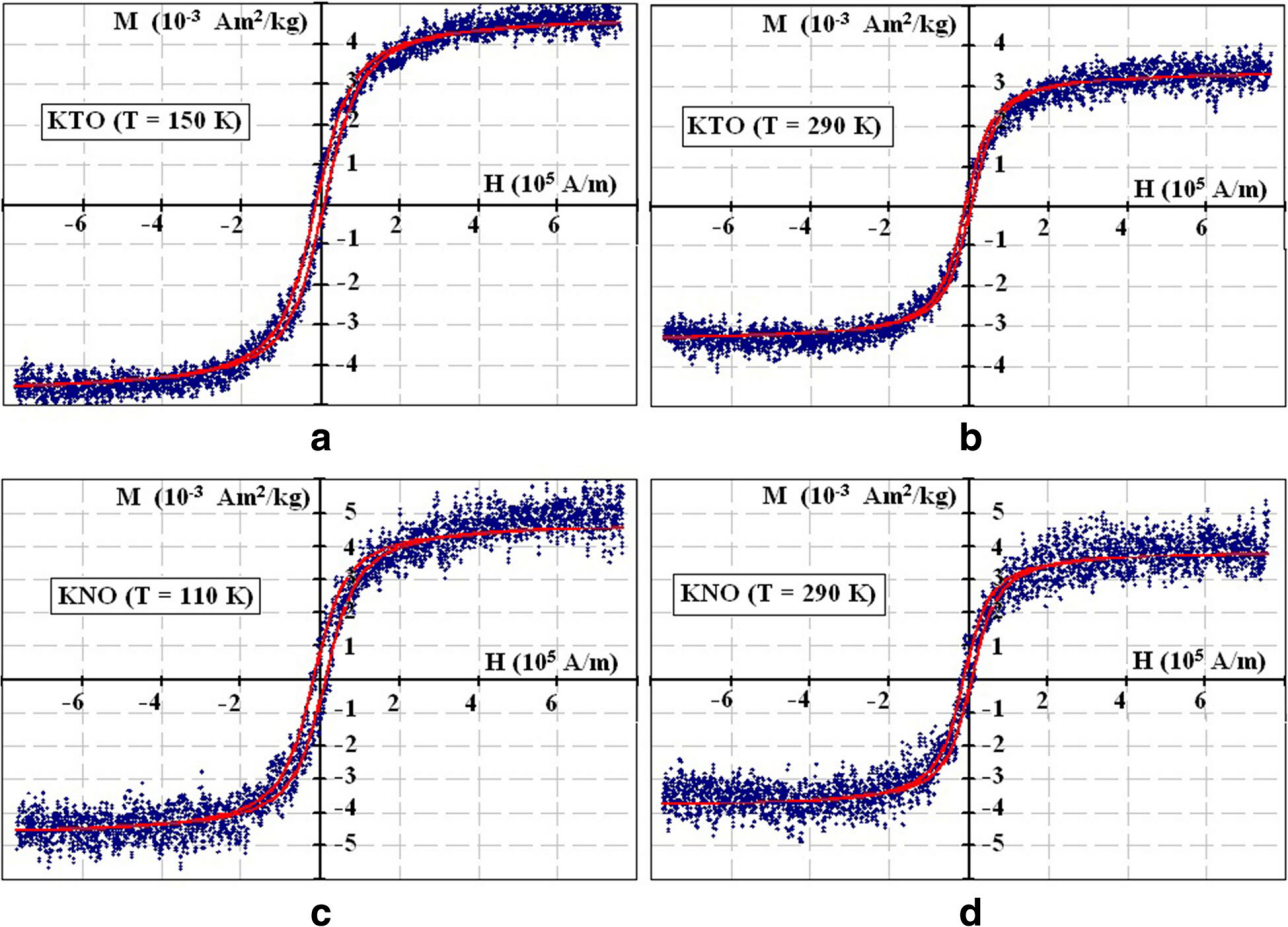
Static magnetization curves. Magnetization curves in KTaO_3_ (**a**, **b**) and KNbO_3_ (**c**, **d**) nanoparticles at *T* = 150, 290, 110, and 290 K. Fitting parameters are listed in Table 1

Here, *M*_*S*_ is the saturation magnetization, *H* is a static magnetic field, and *V*_0_ is the volume of infinite (in fact, closured over a nanoparticle surface) magnetic cluster. Saturation magnetization was defined in accordance with the formula (7) from [20]:

2 $$ {M}_S=\left|{S}_1{N}_1-{S}_2{N}_2\right| $$

where *S*_*1*_ and *S*_*2*_ are V(O) and Fe^3+^ magnetic moments (spins of V(O) and Fe^3+^ are 1/2 and 5/2, respectively), *N*_1_ and *N*_2_ are the numbers of V(O) and Fe, respectively. Given values *N*_*2*_ correspond to the concentrations 0.06 mol.% for KTO and 0.008 mol.% for KNO obtained from elemental analysis, values *N*_*1*_ were determined accordingly. The fitting results are shown in Fig. 3. For a description of the hysteresis loops at low magnetic fields, we used the shift of formula (2) on the value of the coercive field, *H*_*c*_. Parameters for each curve are given in the Table 1.

**Table 1:** Parameters extracted from the fitting of magnetization curves

Parameter	KTaO_3_		KNbO_3_	
*T* (°K)	150	290	110	290
*M*_*s*_ (Am^2^/kg)	4.75 × 10^−3^	3.4 × 10^−3^	4.75 × 10^−3^	3.9 × 10^−3^
*V*_0_ (m^−3^)	9 × 10^−24^	1.7 × 10^−24^	10^−24^	4 × 10^−23^
*H*_*c*_ (A/m)	10^4^	0.7 × 10^4^	1.5 × 10^4^	10^4^
*N*_*1*_ (1/kg)	2.16 × 10^13^	1.6 × 10^13^	3.59 × 10^13^	2.95 × 10^13^
*N*_*2*_ (1/kg)	2.07 × 10^12^	1.33 × 10^12^	2.63 × 10^11^	3.59 × 10^13^

**Fig. 4:**
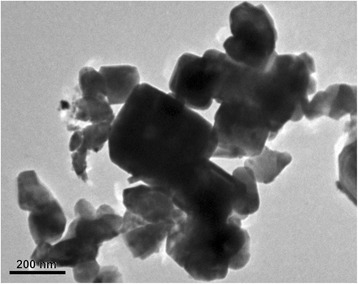
TEM image of the KTaO_3_ nanoparticles

**Fig. 5:**
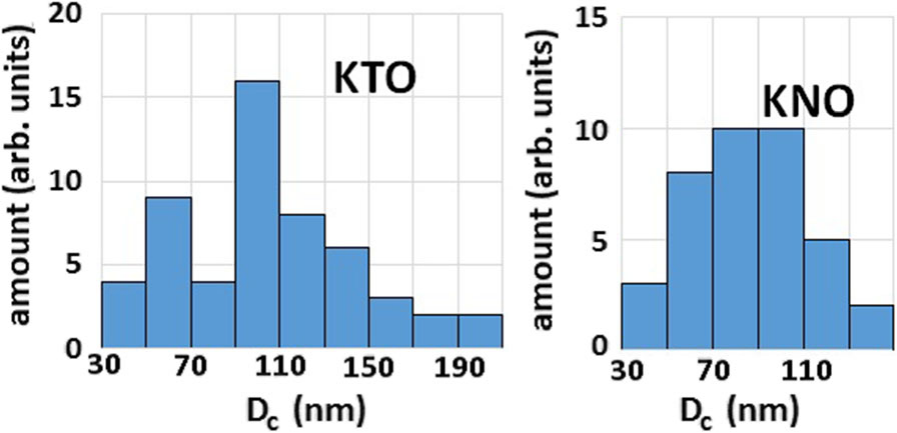
The size distribution of crystallites in KTaO_3_ and KNbO_3_

According to the number of magnetic spins estimated from the EPR data [10, 15], the percentage ratio between the paramagnetic and ferromagnetic subsystems is 14/86 in the nanosized potassium tantalate, and it is 40/60 for potassium niobate. The greater number of paramagnetic centers in potassium niobate is caused by the presence of uncontrolled manganese impurity in addition to the iron impurity. Note that the number of magnetic spins forming ferromagnetic subsystem, obtained from previous experiments, is not sufficient to establish long-range magnetic order in the entire volume of the nanoparticles. Therefore, we assume that the main contribution to the ferromagnetism are magnetic spins, which are near the surface of the particles, namely in the sub-surface layer enriched by defects. As stated in [24], the surface layer enriched by polar defects is about 10 lattice constant thick. On the other hand, the authors of Ref. [25] suggest that the layer enriched by magnetic defects in semi-infinite crystal is 1 lattice constant thick. Given that the complexes Fe^3+^-V(O) are both polar and magnetic defects, we suppose that the near-surface defect layer is of 5 lattice constants in our calculations. In KTaO_3_ and KNbO_3_, the layer corresponds to 2 nm. In order to use the percolation conditions derived in the paper [17], we assume that the distribution of defects is uniform in the near-surface layer. According to transmission electron microscopy (TEM) data (Fig. 4, see also Fig. 1 in Ref. [9] and Fig. 2 in Ref. [26]), the shape of КTaO_3_ and KNbO_3_ nanoparticles can be modeled by a cube. The size distribution of crystallites in each compound obtained from the TEM data is presented in Fig. 5.

**Fig. 6:**
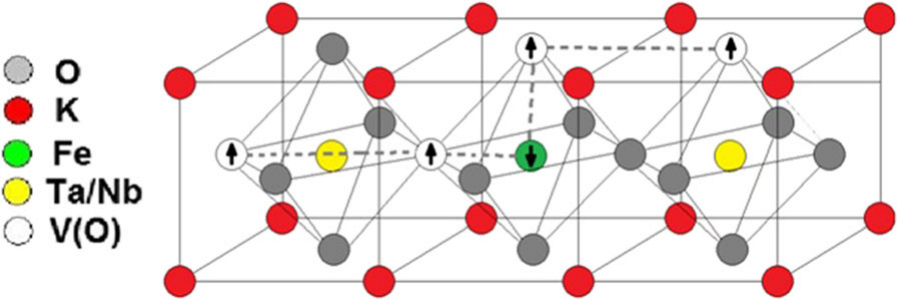
Model of long-range magnetic order between electrons trapped in oxygen vacancies in the non-stoichiometric KTaO_3_/KNbO_3_ compound

Based on experimentally obtained magnetization curves, particularly taking into account the given numbers of V(O) and Fe (see Table 1), we notice the dominant role of the oxygen vacancies in magnetization. At this assumption, an exchange interaction can be initiated by electrons trapped in oxygen vacancies and mediated by magnetic impurity Fe ions. Such interaction has been considered in the model of bound magnetic polarons. This model was first proposed by the authors of [22] to describe the appearance of ferromagnetism in diluted magnetic semiconductors. Schematically, our model is presented in Fig. 6.

### Theoretical Modeling Within Percolation Approach

To determine the critical concentration of total defects, which can lead to the formation of magnetic polarons, we use a percolation approach. Using the criteria of percolation theory, it was shown in the work [17] how the percolation threshold of the long-range magnetic order is established mathematically. Omitting the details of the approaches and principles from Ref. [17], we will focus on the features used in our calculations and describe the general course of the calculation.

**Fig. 7:**
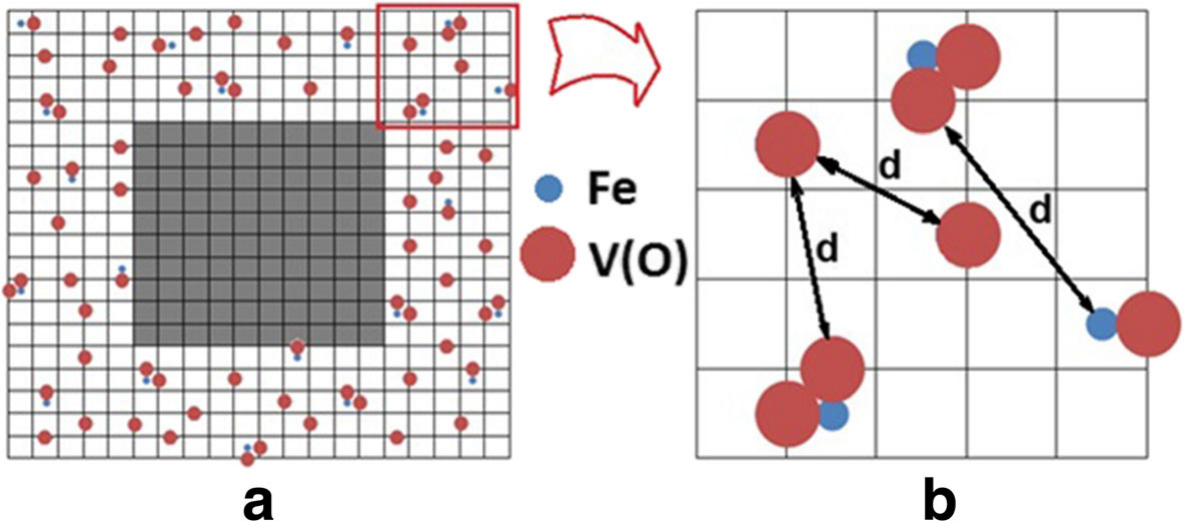
Distribution of defects in the surface layer. **a** *Light area* displays the sub-surface defect layer. *Gray area* reflects the depth of nanoparticles, where defects are almost absent in comparison with the sub-surface layer. Proportionality between the size of sub-surface defect area and defect-free inner core is broken in this figure for more clear representation of the defects distribution in the sub-surface layer. Fe atoms are distributed uniformly in the depth of the defect layer, the oxygen vacancies V(O) are located near Fe atoms. **b** Zoom of the defective layer (**a**); *d* is the distance between defects

In accordance with the TEM data (see Fig. 4), considered KTaO_3_ and KNbO_3_ nanoparticles can be modeled by a cube. The cubic lattice with a 0.4-nm lattice constant was used. In the near-surface layer of 2 nm thickness (that is 5 unit cells) (background is given above, see also Refs. [24] and [25]) with randomly distributed defects, Fe atoms and oxygen vacancies V(O), taking into account that the distribution of iron atoms is uniform. According to the models of axial and rhombic centers (see Fig. 1), the presence of Fe atom in the center of the unit cell (when it substitutes Nb or Ta atoms in the KTaO_3_ or KNbO_3_ lattice) is accompanied by the appearance of V(O) on the edge(s) of the cell. With some probability, one oxygen vacancy V(O) or two vacancies 2 V(O) may occur. The probability is 50% in our case that corresponds to the experimentally determined ratio of the axial (Fe-V(O)) and rhombic (Fe-2 V(O)) centers in KTaO_3_ and KNbO_3_ [9, 10, 15]. The defects were modeled by spheres in our calculations, at that the radiuses of iron ion and oxygen vacancy are *r*(Fe^3+^) = 0.064 nm and *r*(V(O)) = 0.132 nm, respectively. The distance *d* between the defects was defined as the distance between the surface of the spheres and not between their centers. Knowing the coordinates of random defects, namely Fe atoms and V(O) vacancies, we calculate the distance *d* between them. The result of the distribution of defects is shown schematically in Fig. 7.

According to percolation theory, long-range magnetic order in the system occurs as soon as the infinite cluster forms (see, e.g., p.235 in Ref. [17]). The distance between the defects, which form an infinite cluster, and therefore, fulfill the percolation condition (“percolation threshold”), is defined as a percolation radius, *R*_perc_. To determine the percolation radius, the calculation was performed for the nanoparticles of different sizes, to ensure that the percolation condition is actually fulfilled. For the case, the ratio between the number of magnetic defects in the infinite cluster and the total number of magnetic defects in the system remains unchanged in particles of different sizes. The size of the nanoparticles is *D* × *D* × *D* unit cells, where *D* = 20, 30, 40. To determine the dependence of the percolation radius on the concentration of magnetic defects, the calculations were performed for the concentrations presented in the Table 2. Here, *n*_1_ defines the concentration of oxygen vacancies which are not located near Fe atoms, *n*_2_ is the concentration of Fe atoms, and *n* is a total concentration of defects. Note that we perform calculations for two cases. For case I, *n*_1_/*n*_2_ = const and *n* varies. For case II, *n*_2_ = const and *n*_1_ varies.

**Table 2:** Concentrations for which the calculations were performed

*n*_1_/*n*_2_ = const		*n*_2_ = const	
KTO (*n*_1_/*n*_2_ = 13, 49)	KNO (*n*_1_/*n*_2_ = 134, 85)	KTO (*n*_2_ = 0.0422 nm^−3^)	KNO(*n*_2_ = 0.004238 nm^−3^)
*n* (nm^−3^)	*n* (nm^−3^)	*n*_1_ (nm^−3^)	*n*_1_ (nm^−3^)
0.61172547	0.575857	0.499163827	0.564556676
0.489380376	0.460686	0.285236473	0.282278338
0.293628226	0.276412	0.199665531	0.188185559
0.146814113	0.138206	0.15746493	0.141139169
0.058725645	0.055282	0.131358902	0.112911335
0.017617694	0.016585	0.113446324	0.094092779
		0.092437746	0.070569584
		0.079232354	0.056455668
		0.070304764	0.04704639
		0.062395478	0.037637112

In order to determine the percolation radius *R*_perc_, we use the following procedure. For each concentration, we set a certain value *R* that was varied from 0.1 to 5.9 nm with an increment of 0.2 nm. The distance *d* between the defects was compared with the value *R* (for each *R* value). Thus, all magnetic defects are divided into two groups. If the distance *d*_*ij*_ between the nearest *i*th and *j*th defects is less than or equal to the *R*, i.e., *d*_*ij*_ ≤ *R*, we classify these defects to a group, in which there is a magnetic coupling between the defects; otherwise, if *d*_*ij*_ > *R*, we refer such defects to the other group, where the coupling between defects is absent (i.e., broken). As a result of the calculation, we obtain the matrix of *m* × *m*, which elements are Boolean values 1/0 (presence/absence of coupling between the *i*th and *j*th defects). Here, *m* is the number of defects in the sub-surface area in the particle of a certain size. Next, using the principle of Markov chains, we find the magnetic clusters (i.e., aggregates of points {Fe, V (O)}), which indirectly interact. For this purpose, we raised the aforementioned *m* × *m* matrix to the power *m* and get a new matrix *m* × *m*, which elements are Boolean values 1/0 (presence/absence of mediated interaction between the *i*th and *j*th defects). The maximum sum of the matrix line corresponds to the size of the largest cluster for a given particle size. Similar calculations were performed for each value of *R* and the size of the nanoparticles. Obtained results for one the concentration *n* = 0.6117 nm^−3^ are listed in the Table 3. The italics area in the table corresponds to the *R* = *R*_perc_ value that is the percolation radius *R*_perc_ = 1.7 nm. Percolation radius *R*_perc_ was found in accordance with the above-described percolation threshold.

**Table 3:** Number of defects in the infinite cluster for different distances *R* and values *D*

Defect concentration *n* = 0.6117 nm^−3^ (KTO)							
D	*R* (nm)						
	1.1	1.3	1.5	*1.7*	1.9	2.1	2.3
20	14.56%	28.13%	57.83%	*87.54%*	98.39%	99.48%	99.68%
30	8.10%	17.14%	49.12%	*86.82%*	98.12%	99.51%	99.78%
40	4.61%	11.09%	34.12%	*81.33%*	98.14%	99.56%	99.84%

**Fig. 8:**
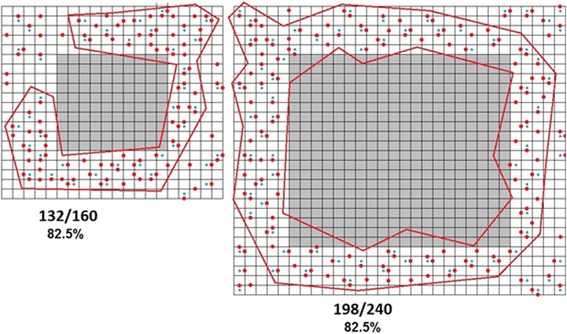
Infinite cluster formation in the particles of different size. The percolation threshold is 82.5%, defined as the percentage ratio of defects in the cluster is not dependent on particle size, as anticipated

Figure 8 schematically shows the formation of the infinite cluster in the particles of different sizes.

## Results and Discussion

**Fig. 9:**
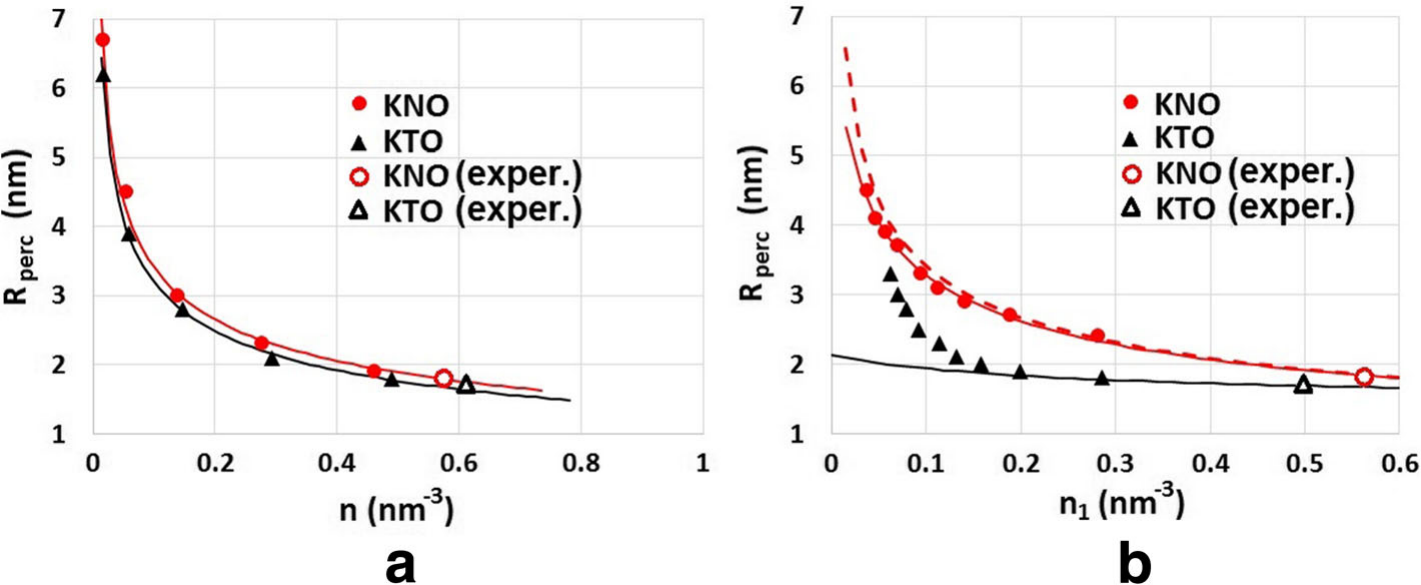
Percolation radius versus the concentration of defects for two cases: **a** *n*_1_/*n*_2_ = const, *n* varies and **b** *n*_2_ = const, *n*_1_ varies. Points are calculated numerically in the framework of our model, solid curves are based on the formula (1). Points marked with labels correspond to the studied materials (KTaO_3_ and KNbO_3_)

Figure 9a shows the dependence of the percolation radius *R*_perc_ on the concentration of defects for the case I, i.e., when *n*_1_/*n*_2_ = const and *n* varies. The solid curves are plotted using the formula

3 $$ {R}_{\mathrm{perc}}=\frac{a}{\sqrt[3]{n}}- b $$

with parameters *a* = 1.6 and 1.7 for KTO and KNO, respectively, and *b* = 0.25 nm for both, KTO and KNO. The first term in the formula (3) is consistent with the equation (4) in Ref. [20] for a percolation radius, ${R}_{\mathrm{perc}}=\frac{a}{\sqrt[3]{n}}.$

The value of *R*_perc_ is a certain critical value, for *R* > *R*_perc_, the infinite cluster will be formed, which is within the necessary condition of the long-range magnetic order appearance in the framework of percolation theory. We assumed that the radius *R*_perc_ is the same for the pairs Fe-Fe, V(O)-V(O), and V(O)-Fe. However, based on the calculated results and experimental facts, we can state that the radius *R*_perc_ cannot be less than 1.6 nm for KTaO_3_ and 1.7 nm for KNbO_3_.

Let us consider the formula (3) in more details. Physical meaning of the expression $1/\sqrt[3]{n}$ is the distance between defects participating in the magnetic exchange for the appearance of long-range ordering. The value *a* can be both less and more than 1. When the radius *R*_perc_ corresponds to the average distance between defects, the value *a* = 1, and so ${R}_{\mathrm{perc}}=1/\sqrt[3]{n}$. When the number of random magnetic couplings, which are formed by the interaction between defects at a distance less than the average, is sufficient for the formation of infinite cluster, value *a* is less than 1. For example, *a* = 0.86 in the work [17]. It turns out that the *R*_perc_ is 1.6–1.7 times higher than the average distance between defects in our case. This can be explained as follows.

The value of the average distance between defects makes sense for a uniform distribution of defects in the sub-surface layer. The distribution of Fe atoms is regarded as quasi-uniform, but taking into account the fact that the Fe atoms are placed in the center of the cell, their positions are discrete spatial coordinates, which also affects on the value *a* in the Eq.3, and the location of oxygen vacancies V(O) is not uniform because they are always nearby Fe atoms (see Fig. 6). Thus, the distance between Fe atoms is much greater than the distance between the Fe atom and the oxygen vacancy V(O). Therefore, the radius *R*_perc_ actually does not depend on the average distance between all defects, but it depends on the average distance between defects in different unit cells (this may be the distance between the Fe-Fe, V(O)-V(O), V(O)-Fe). The presence of oxygen vacancies near the Fe atoms slightly reduces the distance between the defective cells due to the smallness of the V(O)-Fe distance compared to Fe-Fe distance. That means that in this case, we are actually dealing with the average distance between Fe atoms.

**Fig. 10:**
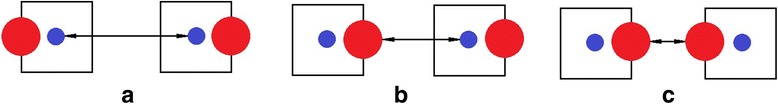
Reducing of *R*_perc_  for interaction between different defects (**a**-**c**) in comparison with a uniform distribution of Fe (*blue*) atoms is caused by the presence of oxygen vacancies V(O) (*red*)

Subtraction of the second component *b* = 0.25 nm in Eq.(3) can be explained as follows. Typically, the distance between defects in this equation is defined as the distance between the centers of defects, if a defect is simulated as a point. In our calculations, *R*_perc_ is defined as the distance between the surfaces of the sphere in which we placed the defect(s). So, the value R_perc_, estimated as the distance between the surfaces of the spheres, is different from the *R*_perc_ estimated as the distance between the centers at least on the sum of two radii of interacting defects. The sum can vary from 0.128 nm for Fe-Fe interaction (Fig. 10a) to 0.264 nm for V(O)-V(O) interaction (Fig. 10c). In addition, since the distance between the Fe atom and the nearest vacancy V(O) is much less than the calculated one, required to establish a coupling between defects, in certain cases, *R*_perc_ can be defined as the distance V(O)-Fe or V(O)-V(O) from different cells (see Fig 10b, c). When the number of V(O) increases, the number of cases presented in Fig. 10b, c also increases, respectively. Accordingly, the calculated value of *b =* 0.25 nm indicates that mainly an exchange interaction occurs directly between the electrons trapped in oxygen vacancies.

Figure 9b shows the dependence of the percolation radius *R*_perc_ on the concentration of defects for the case II, i.e., when *n*_2_ = const and *n*_1_ varies. Here, the influence of Fe atoms becomes greater as *n*_1_ decreases. This is more pronounced for KTO (black solid curve in Fig. 9b). Nevertheless, we tried to fit the calculated data using the formula (3) with parameters *a* = 0.66 and *b* = 0.15 nm. The value of *b* indicates that relative amount of direct V(O)-V(O) interactions decreases, while the amount of indirect interactions via Fe atoms increases.

## Conclusions

We consider microscopic mechanism that leads to the emerging of ferromagnetic ordering in ferroelectric KTaO_3_ and KNbO_3_ nanoparticles. Our approach is based on the magnetic percolation theory. It describes the formation of surface magnetic polarons in which an exchange interaction between charge carriers, i.e., electrons, trapped in oxygen vacancies occurs directly or indirectly via magnetic Fe atoms.

The dependence of radius *R*_perc_ on the total concentration *n* of defects at constant *n*_1_/*n*_2_ ratio (*n*_1_ is the concentration of oxygen vacancies and *n*_2_ is the concentration of Fe atoms) is determined in the framework of percolation theory. Appeared that the dependence is well-described by the formula ${R}_{\mathrm{perc}}=\frac{a}{\sqrt[3]{n}}- b$, where the values of the parameters *a* = 1.6 and 1.7 for KTO and KNO, respectively, and *b* = 0.25 nm for both, KTO and KNO. It is found out that real magnetic percolation radius cannot be smaller than 1.6 nm for KTaO_3_ and 1.7 nm for KNbO_3_.

Using the calculated critical concentration of magnetic defects, experimentally measured magnetic hysteresis loops are well described by two shifted Langeven-type formulas. Magnetization saturation value *M*_*S*_ depends on the magnetic moments of oxygen vacancies V(O) and Fe^3+^ ions and their amounts *N*_1_ and *N*_2_, respectively, in the infinite cluster as *M*_*S*_ = |*S*_1_ *N*_1_ − *S*_2_ *N*_2_|, that is in a complete agreement with Ref. [20].

Theoretical calculations adequately describe the experimental results obtained in ferroelectric KTaO_3_ and KNbO_3_ nanoparticles synthesized by oxidation of metallic tantalum in molten potassium nitrate with the addition of potassium hydroxide, which exhibit a weak ferromagnetism, while these compounds are nonmagnetic in a bulk.

## Abbreviations

AFM: Atomic force microscopy
MESP: Cobalt-coated magnetic force probes
MFM: Magnetic force microscopy
TEM: Transmission electron microscopy
